# The Prevalence of Tuberculosis in Zambia: Results from the First National TB Prevalence Survey, 2013–2014

**DOI:** 10.1371/journal.pone.0146392

**Published:** 2016-01-15

**Authors:** Nathan Kapata, Pascalina Chanda-Kapata, William Ngosa, Mine Metitiri, Eveline Klinkenberg, Nico Kalisvaart, Veronica Sunkutu, Aaron Shibemba, Chishala Chabala, Gershom Chongwe, Mathias Tembo, Lutinala Mulenga, Grace Mbulo, Patrick Katemangwe, Sandra Sakala, Elizabeth Chizema-Kawesha, Felix Masiye, George Sinyangwe, Ikushi Onozaki, Peter Mwaba, Davy Chikamata, Alimuddin Zumla, Martin P. Grobusch

**Affiliations:** 1 National TB and Leprosy Control Program, Lusaka, Zambia; 2 Ministry of Health Headquarters, Lusaka, Zambia; 3 KNCV Tuberculosis Foundation, The Hague, the Netherlands; 4 Department of Global Health, Academic Medical Centre, University of Amsterdam, Amsterdam Institute for Global Health and Development, Amsterdam, The Netherlands; 5 University Teaching Hospital, Lusaka, Zambia; 6 Department of Public Health, University of Zambia, Lusaka, Zambia; 7 Tropical Diseases Research Centre, Ndola, Zambia; 8 Chest Diseases Laboratory, Ministry of Health, Lusaka, Zambia; 9 Department of Economics, School of Humanities and Social Sciences, University of Zambia, Lusaka, Zambia; 10 United States Agency for International Development, Country Mission, Lusaka, Zambia; 11 Global Tuberculosis Programme, World Health Organisation, Geneva, Switzerland; 12 Ministry of Home Affairs headquarters, Lusaka, Zambia; 13 Division of Infection and Immunity, Department of Infection, University College London, London, United Kingdom; 14 Centre for Tropical Medicine and Travel Medicine, Department of Infectious Diseases, Academic Medical Centre, University of Amsterdam, Amsterdam, The Netherlands; Brighton and Sussex Medical School, UNITED KINGDOM

## Abstract

**Background:**

Tuberculosis in Zambia is a major public health problem, however the country does not have reliable baseline data on the TB prevalence for impact measurement; therefore it was among the priority countries identified by the World Health Organization to conduct a national TB prevalence survey

**Objective:**

To estimate the prevalence of tuberculosis among the adult Zambian population aged 15 years and above, in 2013–2014.

**Methods:**

A cross-sectional population-based survey was conducted in 66 clusters across all the 10 provinces of Zambia. Eligible participants aged 15 years and above were screened for TB symptoms, had a chest x-ray (CXR) performed and were offered an HIV test. Participants with TB symptoms and/or CXR abnormality underwent an in-depth interview and submitted one spot- and one morning sputum sample for smear microscopy and liquid culture. Digital data collection methods were used throughout the process.

**Results:**

Of the 98,458 individuals who were enumerated, 54,830 (55.7%) were eligible to participate, and 46,099 (84.1%) participated. Of those who participated, 45,633/46,099 (99%) were screened by both symptom assessment and chest x-ray, while 466/46,099 (1.01%) were screened by interview only. 6,708 (14.6%) were eligible to submit sputum and 6,154/6,708 (91.7%) of them submitted at least one specimen for examination. MTB cases identified were 265/6,123 (4.3%). The estimated national adult prevalence of smear, culture and bacteriologically confirmed TB was 319/100,000 (232-406/100,000); 568/100,000 (440-697/100,000); and 638/100,000 (502-774/100,000) population, respectively. The risk of having TB was five times higher in the HIV positive than HIV negative individuals. The TB prevalence for all forms was estimated to be 455 /100,000 population for all age groups.

**Conclusion:**

The prevalence of tuberculosis in Zambia was higher than previously estimated. Innovative approaches are required to accelerate the control of TB.

## Introduction

The burden of tuberculosis (TB) in Zambia is among the highest in the African region and in 2013 the prevalence was estimated at 388/100,000 population according to the World Health Organization (WHO) and an estimated incidence rate of 427/ 100 000 population; TB is one of the major public health problems in the country with a notification rate of 289/ 100 000 population of all forms of TB in 2012. Zambia was one of the 22 high priority countries selected by the World Health Organization (WHO) to undertake a national TB prevalence survey [1], because it met the criteria as defined by the WHO Global Task Force on TB Impact Measurement [2]. Historically, there have been no national TB prevalence surveys that have been conducted in Zambia. Therefore, the country lacked baseline data on the prevalence of tuberculosis disease (TB). The WHO recommends implementation of population-based prevalence surveys to estimate the prevalence of TB for baseline and consequently impact assessment to measure progress [2, 3].

Currently, Zambia is relying on routine surveillance data from health facilities collected through the national TB control program, to measure progress towards TB control targets. This data has limitations such as under-reporting, and it does not provide information on the number of undetected cases in the community and on those individuals seeking care outside of the public sector. The HIV pandemic and poverty in the Country may have also led to increase in the TB burden; furthermore, routine surveillance data may have gaps due to recording and reporting bias [4]. Therefore, a nationally representative population-based survey was conducted to estimate the burden of disease to inform policy makers and to provide baseline data for measurement of program achievements.

The aim of the survey was to estimate the adult (15 years and older) prevalence of sputum and culture positive pulmonary tuberculosis (PTB). The other objectives were to study the health seeking behaviour and the association between household income and TB disease among the TB cases.

## Materials and Methods

The study was a nationally representative cross sectional community based survey among households in selected clusters. A sample size of approximately 54,400 adults (aged 15 years or more) was targeted to estimate the prevalence of pulmonary TB based on the following assumptions: Prevalence of smear-positive pulmonary TB = 199/100,000 population [5] with a precision of 25%; a design effect of 1.5 and expected response rate of 85%.

The 66 clusters were selected in 49 districts in all the 10 provinces of Zambia. These were randomly selected, following a two-stage probability proportion-to-size (PPS) sampling strategy. The average cluster size was 825 inhabitants. The sampling frame consisted of a list of Census Supervisory Areas (CSAs) as primary sampling units (PSUs) from all administrative areas.

Data collection was implemented using three field teams between September 2013 and July 2014. The country was zoned into three regions and each team was assigned a specific region.

In each cluster, the census team conducted household visits to register all eligible household members, and to administer a social economic questionnaire to household heads. The electronic census register and socioeconomic questionnaire were pre-loaded on the Personal Digital Assistants (PDAs) before the field teams started to conduct the survey in the cluster.

All eligible household members (individuals meeting the inclusion criteria and who had slept in the house during the previous 24 hours before the census team visited the households, irrespective of nationality) were invited to a central survey site located within the cluster. The inclusion criteria was based on individuals living in households within the selected clusters who were 15 years or older; were able to give informed consent and had slept in the household in the previous 24 hours prior to survey team visit. The individuals who met the exclusion criteria were those aged less than 15 years or were unable to understand the information given to them because they were deaf and a sign language interpreter was not at hand at time of recruitment; or they had speech impediments and thus could not communicate; or if they were incapacitated by disease such that they could not understand the information given to them. Others who were excluded were people that were considered to be living in institutional settings, such as prisons, boarding schools, military camps, Hotels and Lodges. Individuals who were unable to provide consent or assent were also excluded.

The definitions of TB as per the national protocol were: (i) Acid fast bacilli (AFB) positive smear which was a Fluorochrome-stained smear with at least one acid fast bacilli in 40 fields (ii) Smear positive pulmonary TB patient was a patient with at least one specimen positive for AFB by smear microscopy (iii) A Culture positive pulmonary TB patient was a patient with Mycobacteria Growth indicator Tube positive showing presence of AFB and coding By Ziehl-Neelsen (ZN) stain and identified with TAUNS (capillia) test as Mycobacterium tuberculosis complex within the maximum incubation period of 42 days. (iv) A bacteriologically confirmed pulmonary TB patient was a patient with smear-positive and/or culture-positive pulmonary TB (or Xpert MTB/RIF positive). (v) New smear-positive pulmonary tuberculosis patient was a smear-positive pulmonary TB patient having no history of treatment for tuberculosis or had taken anti-tuberculosis drug for less than a month.

Consenting eligible participants were screened for TB symptoms using a structured questionnaire and underwent chest x-ray (CXR) screening using mobile digital CXR units to determine their eligibility to submit sputum for laboratory investigation (presumptive TB patients). A normal chest X ray was defined as an X-ray showing clear lung fields and no abnormality detected whereas an abnormal chest X ray was defined as one showing any lung abnormality (e.g. opacities, cavitation, fibrosis, pleural effusion, calcification, any unexplained or suspicious shadow) and heart abnormalities detected on interpretation by a qualified medical officer. The Chest X-rays were initially read in the field immediately by Medical officers and then images were electronically transmitted to the central radiology unit at the University Teaching Hospital where they were re-read by a specialised Radiologist. They was a panel of four specialized radiologists who were at all at post graduate level of qualification and each had at least more than ten years’ experience involved in reading the X-rays.

The PDAs were programmed to automatically identify presumptive TB patients based on the outcome of either the symptom-screening interview or field CXR examination. Household members reporting to have any of the following symptoms: cough for ≥ 2 weeks, *or* fever for ≥ 2 weeks, *or* chest pains for ≥ 2 weeks, or those with abnormal shadows on chest CXR or indeterminate CXR findings were requested to submit two sputum samples, one on the spot and one morning sample. After the collection in the field, the clearly labelled sputum specimen’s containers were sealed by tightening the screw cap and cleaning with bleach any contamination on exterior of the container. Each container was packed in a zip-locked leak-proof plastic bag with biohazard label. The sputum specimens were kept cool and stored a cool box with ice packs and were transported to the laboratories under same conditions in a refrigerated cool box or one with ice packs to reduce culture contamination rates. All sputum specimens were transported from the field within three or less days (five days was the maximum transport time). A courier system was in place to ensure that the specimens were delivered within the specified timelines. The specimens were processed within 5 days from the date of collection.

The samples were examined for AFB smear using the Auramine-Phenol method and liquid culture (BACTEC ^TM^ MGIT ^TM^ 960) at any of the three central reference laboratories (CRLs). Each sample was digested and decontaminated using the Sodium Hydroxide N-acetyl-L-Cysteine (4%NaOH-NALC) method and inoculated into one MGIT media and incubated in automated MGIT machines. The remaining deposits were used for concentrated AFB smear microscopy using Fluorescent Microscopy technique. The inoculum was plated on a non-selective blood agar plate and incubated for 24 hours. The remaining inoculum was stored at 4°C and only discarded after the outcome of the contamination check. Culture reading was performed daily and confirmation of positive Mycobacteria tuberculosis growth was done by Acid fastness on ZN stain and Capillia Test. All isolates, both *M*. *tuberculosis* and Non-Tuberculous Mycobacteria, were stored at -70°C.

Additionally, the Xpert MTB/RIF assay (Cepheid, Sunnyvale, CA, USA) was performed to confirm MTB positivity for all smear positives cases. Samples from cases with culture indeterminate and CXR suggestive of TB were also subjected to XpertMTB/RIF assay (Xpert).

Quality assurance for AFB Smear Microscopy was conducted in such a way that all the slides were examined by trained biomedical technical personnel meeting national AFB smear microscopy proficiency test standards. The slides were stored serially after examination and were prepared for blinded re-checking of a random sample that was conducted at the end of each cluster operation. A negative and positive (+1) control for every 24 slides stained was included. Internal quality control for culture was done using the reference strain H37Rv as a positive control and sterile phosphate buffered solution as a negative control. The controls were included in every batch of 14 samples run. 3 ml of suspension of 0.5 McFarland’s H37Rv bacterial suspension and 3 ml of sterile PBS were included in each batch of samples run. The participants who were eligible to submit sputum underwent an in-depth interview, using an electronic structured questionnaire. After completing TB related procedures, all consenting survey participants were also offered HIV counselling and testing on an opt-out basis. If the participant agreed to participate, the HIV testing and counselling was conducted in accordance with national algorithm. Participants who consented to HIV testing had their blood collected through a finger prick and blood was placed directly onto the screening rapid test strip (Determine™). Pre-test and post-test counselling was provided to all participants who choose to know their results. Pre-test counselling was done prior to testing. The counselling was performed by trained nurse counsellors. Participants had an option to be informed or not on the results of their HIV test. If the test was non-reactive, a negative result was communicated to the participant. However, if the test was reactive, the participant was asked to give a second blood sample from another finger prick for a confirmatory test (Uni-Gold™ Recombigen®). If the second test was reactive, the participant was informed of the positive status, appropriately counselled and referred for further management to the nearest health facility which was already identified in the vicinity of each and every survey cluster. If the second test was non-reactive, the result was considered indeterminate and the participant was advised to visit the nearest health facility for a further testing after six weeks.

Eligible adults who failed to show up at the survey site for screening, were followed up at household level by Community Health Volunteers (CHVs). During the follow up visit, individuals found to be either very old, sick or physically incapable of going to the survey site were transported by the survey team’s vehicle.

All cases found to have TB according to the national TB treatment guidelines were referred to the nearest health facility for further evaluation and treatment. The survey coordinator was responsible for notifying TB cases to the provincial and district TB officers in the clusters for further case management.

The survey generated mostly digital data with the exception of participant invitation cards, consent forms and hard copy QA questionnaires. A unique Personal Identification Number (PIN) was used in all the stages of data collection and data management. The PIN was converted into a barcode which were used on all forms, registers, specimen containers, results and in digital data files to identify each survey participant.

Data files from the field (on PDAs), the CRLs and the CXR were stored electronically, cleaned and validated separately before being merged in the final MS Access database at the data management unit (DMU). The merge key was the PIN which had been entered in the separate data files from the field, the CRLs and the CXR by reading the barcode of the participant.

Case definition meetings were held every last week of the month to consider all cases with a complete set of results. The case definition meetings were done making sure that all areas of expertise were available to undertake a case-by-case review of the results so as to classify the survey participants using a predetermined case definition framework.

Data was analysed using the STATA software package version 12. The first stage in the analysis focused on describing eligibility, enrolment and participants by age, sex, and province and wealth tertiles. Wealth tertiles were generated by principal components analysis on a standard list of household assets and access to basic amenities. Subsequently the outcome of screening (interview and x-ray film) and sputum testing was described, disaggregating by key characteristics namely sex, age, and type of symptoms or x-ray abnormality, setting and education level. The core analysis focused on estimating the prevalence of pulmonary TB in the adult population. The recommended 3-model approach as proposed by the WHO Global Task Force on prevalence surveys was applied, in line with new guidance on best practice methods based on Floyd et al [6].

Using a two-step approach and three different models, a robust estimate of the TB prevalence was made. As a first step a simple cluster level analysis was done whereby the prevalence rates were calculated at cluster level and then combined to one single point estimate with confidence boundaries. The second step was an individual level model analysis whereby three different logistic regression models were applied, in this article only model 3 results are presented. In Model 3, multiple missing value imputation was done only for those eligible for sputum examination that missed data on outcome. All participants screened out were considered not having TB. In a second step, using inverse probability weighting extrapolation was done to represent all eligible individuals.

### Ethical approval

The study protocol was cleared by the University of Zambia Biomedical Research Ethics Committee (UNZABREC) No: 020-08-12. Authority to conduct the survey was sought in line with the existing national guidelines. Written informed consent was obtained from all individuals who agreed to participate in the survey. For minors, both the assent of the minor and the consent of the next of kin or caretakers or guardian was obtained in writing for each participant aged 15–17 years. The institutional review board (IRB) approved this consent procedure. All the consent or assent forms were recorded on standard forms which were developed for the study and these were filed in lockable cabinets at the end of each cluster operation.

## Results

**Fig 1:**
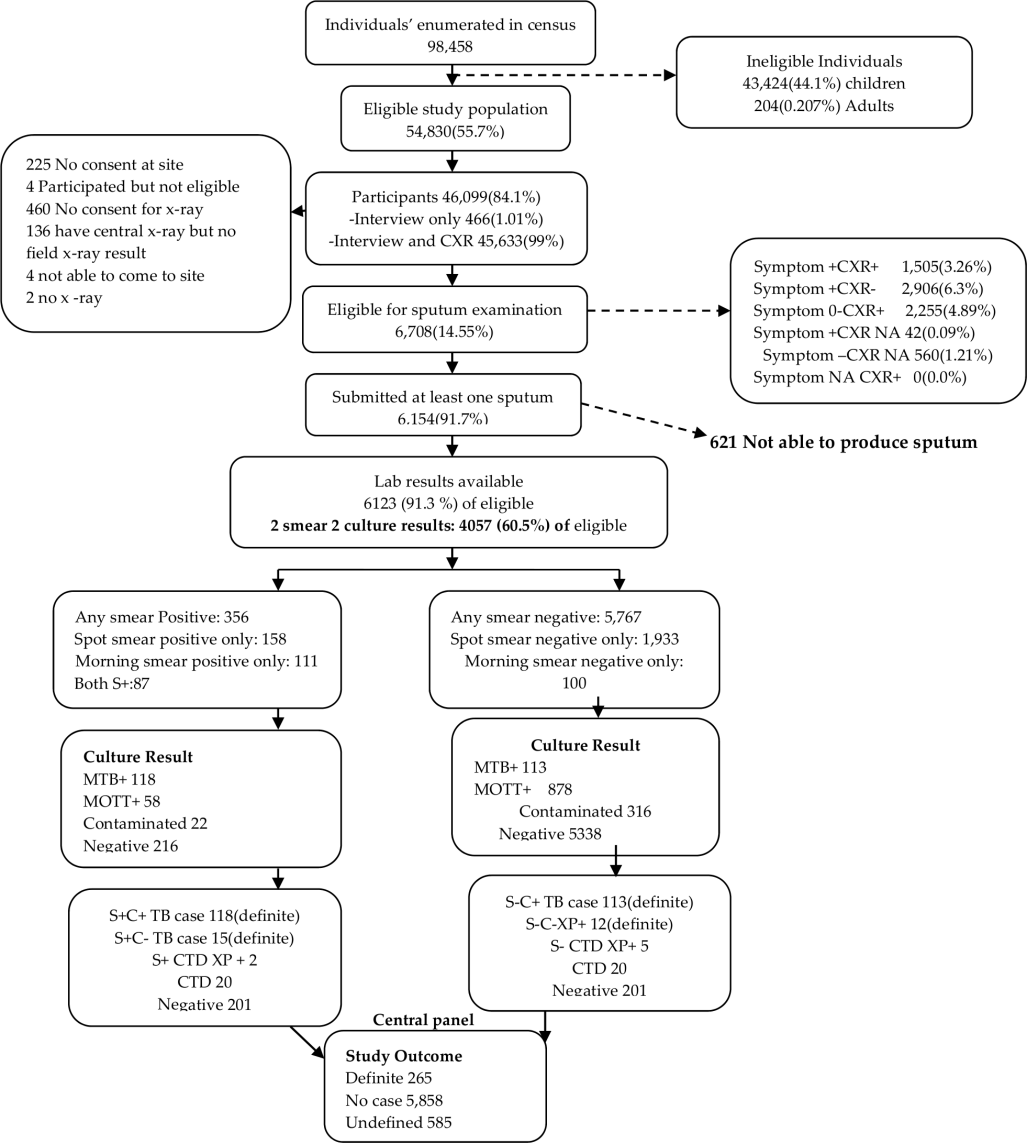
Survey data flow. CXR, Chest X-rays read; Symptom+ CXR+, Symptoms present and Chest X-ray abnormality detected; Symptom+ CXR-, Symptoms present and Chest X-ray abnormality not detected; Symptom- CXR+, Symptoms absent and Chest X-ray abnormality detected; Symptom+ CXR NA, Symptoms present and Chest X-ray not applicable; Symptom- CXR NA, Symptoms absent and Chest X-ray not applicable; Symptom NA CXR+, Symptoms not applicable and Chest X-ray abnormality detected; MTB+, positive for Mycobacterium Tuberculosis; MOTT+, positive for Mycobacterium Other Than Tuberculosis; C+, Culture positive; C-, Culture negative; S+, Sputum smear positive; S-, Sputum smear negative; XP+, Xpert MTB/RIF result positive for MTB; CTD, Contaminated.

From the initial census, 98,458 participants were enumerated in the 66 clusters. Of these, 54,830 (55.7%) were eligible to participate and 46, 099 (84.1%) participated as illustrated in Fig 1.

There were more rural (65.2%, n = 30,042) than urban (34.8%, n = 16,057) participants. The median age of the eligible participants was 32 years (IQR; 22 to 47) with more females (56.4%) than males (43.6%). All the 46,099 survey participants underwent symptom screening, while 45,633 (99.0%) were screened by chest x-ray. Four hundred and sixty six (1.01%) had an interview only; and 45, 633 (99%) had both interview and chest x-ray screening performed. Participants who were found to be positive on symptom screening were 4,453/46,099 (9.7%) while 3,758/45,633 (8.24%) had abnormal chest x-rays and 1,505/4,453 (33.8%) had both symptom positive and chest x-ray abnormal.

**Fig 2:**
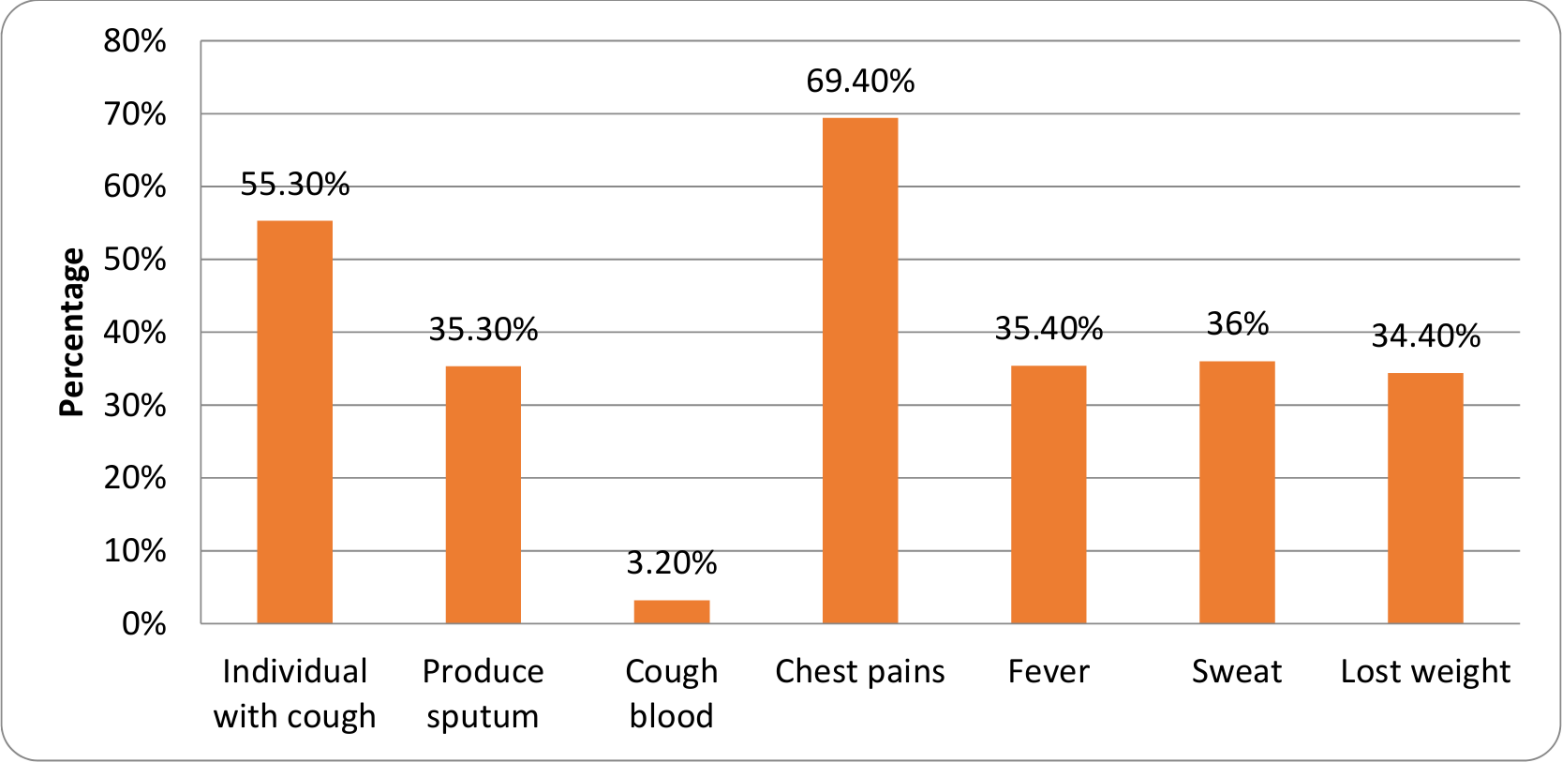
Reported signs and symptoms among presumptive TB participants. Proportions (Percentage) of the different signs and symptoms of the presumptive TB participants.

The majority of the presumptive TB participants (CXR and Symptom) reported at least a history of chest pains (4,653, 69.4%) followed by cough (3,708, 55.3%), night sweats (2,418, 36.0%), fever (2,375, 35.4%), sputum production (2,369, 35.3%), and weight loss (2,306, 34.4%), respectively, as shown in Fig 2.

Based on the results of the symptom and or the chest x-ray screening, 6,708 (14.6%) of the survey participants were eligible to submit sputum sample (presumptive TB patients) for laboratory investigations. Of those eligible for sputum submission, 6154 (91.7%) submitted at least one sample; 6087/6708 (90.7%) at least spot; 4,124/6708 (61.48%) at least morning and, 4,057/6087 (66.65%) submitted both spot and morning sample.

Out of the 6,154 (91.7%) participants who submitted at least one specimen, 265 MTB cases were identified and defined as definite MTB cases by the expert panel. Of these, 135 (50.9%) were smear positive and 130 (49.1%) were smear negative. 45% (118) of the cases were culture and smear positive; 43% (113) were culture positive and smear negative; 39 (0.64%) of the 6123 available laboratory results were Xpert positive for MTB as outlined in Table 1.

**Table 1:** Smear, Culture and Xpert MTB/Rif results (absolute numbers of available laboratory results).

		XpertMTB/Rif				
Smear	Culture	MTB (Positive)	Negative	Not tested	Not applicable	TOTAL
Positive	MTB	4	2	112	0	**118**
	MOTT	8	39	0	4	**51**
	Negative	7	142	0	16	**165**
	Contaminated	2	20	0	0	**22**
	**Total**	**21**	**203**	**112**	**20**	**356**
Negative	MTB	1	0	0	0	**1**
	MOTT	3	95	n/a	4	**102**
	Negative	9	66	n/a	5547	**5622**
	Contaminated	5	32	n/a	5	**42**
	**Total**	**18**	**193**	n/a	**5556**	**5767**
*TOTAL	** **	**39**	**396**	**112**	**5576**	**6123**

MTB, Mycobacterium Tuberculosis; MOTT, Mycobacteria Other Than Tuberculosis; n/a, not applicable.

*TOTAL, Overall total of available laboratory results.

The samples from 585/6708 (8.72%) participants were not processed at the laboratory because they were either inadequate or wrong specimen.

Additionally, 61.75% (161/265) of the survey cases were symptomatic (i.e fever, cough or chest pain lasting two weeks or more) and 82.64% (219/265) had an abnormal CXR. As shown in Table 2, there were 97% symptomatic survey cases not on anti-TB treatment at the time of the survey; and 49.7% of the cases that were not on treatment had sought care for their illness. Of the 156 symptomatic TB cases not on treatment but who sought care; 93% (74) had sought care from a public health facility, 4% (3) from a private facility and another 4% (3) from either a traditional healer of faith-based organization. Of the 7 TB cases (0.3%) who were already on treatment, five were treated at a public facility, none at a private clinic and two from other facility. Furthermore, 15% of the survey cases reported that a member of their household had been treated for TB before.

**Table 2:** Health seeking and treatment status of survey cases.

Variable	N	%
Total number of TB survey cases	265	100
Number of TB survey cases who were not on treatment at the time of the interview	258	97.4
Number of TB cases detected by the survey who were already on treatment	7	0.3
Number of TB survey cases who were symptomatic	161	60.6
Number of *symptomatic* TB survey cases not on treatment at the time of the interview	156	96.9
Number of *symptomatic* TB cases not on treatment but who sought care	80	49.7
Number of *symptomatic* TB survey cases not on treatment who sought care at a public facility	74	94
Number of *symptomatic* TB survey cases not on treatment who sought care at a private facility	3	4
Number of *symptomatic* TB survey cases not on treatment who sought care at other facility	3	4

Non-tuberculous mycobacteria (NTM) were found in 936/6,123 (15.3%) of the individuals whose samples were sent for culture. 13/936 individuals with NTM had MTB co-infection. Of the 923 NTM participants without MTB, 872 were smear negative and 51 were smear positive.

Of the 46,099 TB survey participants 44,761 (97.1%) underwent HIV counselling, out of which 30,584 (68.3%) were tested. The crude prevalence of HIV among survey participants was 6.8% (2062/30,584).

After modelling the 265/46,099 MTB cases identified, the prevalence of smear-positive TB was estimated at 319 per 100,000 adult population, and that of culture-positive TB was 568 per 100,000 population; while bacteriologically confirmed TB prevalence was 638 per 100,000 adult population as shown in Table 3. The prevalence of smear, culture and bacteriologically confirmed TB was higher in males, the HIV positive, those in the age group 35–44 years and the urban participants.

**Table 3:** Smear, Culture and Bacteriological confirmed TB Prevalence.

			Smear			Culture			Bacteriological		
Variable	Number of Survey Participants	Absolute number of survey cases	Prevalence per 100,000	95% CI	p-value	Prevalence per 100,000	95% CI	P-value	Prevalence per 100,000	95% CI	P-value
Overall	46,099	265	319	232–406		568	440–679		638	502–774	
Setting											
Rural	30,042	120	187	130–243		404	295–513		460	344–577	
Urban	16,057	145	583	391–775	<0.001	897	632–1163	<0.001	993	714–1273	<0.001
Sex											
Male	19,457	152	445	309–580		726	548–904		833	641–1024	
Female	26,642	113	221	139–303	<0.001	446	316–576	<0.001	487	353–621	<0.001
Age (Years)											
15–24	14,702	32	154	71–236		255	129–381		285	157–412	
25–34	10,376	66	422	245–599	<0.001	572	362–783	<0.001	664	337–891	<0.001
35–44	8,168	68	496	315–676	<0.001	841	576–1106	<0.001	947	660–1237	<0.001
45–54	5,369	45	323	139–507	0.017	822	517–1127	<0.001	926	611–1240	<0.001
55–64	3,741	23	333	149–517	0.284	663	360–966	0.013	708	401–1013	0.005
65+	3,743	31	288	91–485	0.006	809	452–1167	0.001	876	535–1218	<0.001
HIV Status											
Negative	28,429	98	182	129–236		336	249–422		387	294–480	
Positive	2,062	36	887	424–1350	<0.001	1675	978–2371	<0.001	1726	1029–2423	<0.001
Unknown	14,266	131	499	324–675	<0.001	857	615–1099	<0.001	964	704–1225	<0.001

By socioeconomic status, the burden of TB was similar in the rural areas while in the urban areas; the burden of TB was higher among participants from the lower economic status than those in the higher wealth status as shown in Table 4.

**Table 4:** TB prevalence by socioeconomic status for rural and urban.

			Variable							
Wealth status		Smear			Culture			Bacteriological		
	*Number of survey cases*	*Prevalence per 100*,*000*	95% CI	*P-value*	*Prevalence per 100*,*000*	95% CI	*P-value*	*Prevalence per 100*,*000*	95% CI	*P-value*
**Rural Wealth**	109									
Lowest	42	205	99–310		422	240–603		483	294–672	
Middle	45	138	67–209	<0.001	320	196–445	<0.001	364	224–505	<0.001
Highest	22	248	151–345	<0.001	528	358–698	<0.001	610	423–797	<0.001
**Urban Wealth**	123									
Lowest	7	729	412–1046		1141	685–1597		1208	750–1666	
Middle	21	763	468–1057	<0.001	1098	785–1410	<0.001	1251	911–1592	<0.001
Highest	104	359	183–535	<0.001	521	319–723	<0.001	603	386–820	<0.001

## Discussion

This is the first nationally representative survey to estimate the burden of TB in Zambia. It is also the first fully digital national TB prevalence survey conducted world-wide to the best of our knowledge. Additionally, it is the first national TB survey to concurrently test for HIV among the survey participants. The survey was conducted in accordance with the WHO guidelines [7] on conducting TB prevalence surveys. The participation rate of 84% was by 1% undercutting the lower threshold that was aimed at 85%. The slightly lower participation rate was probably due to the use of large primary sampling units, that is, the CSAs instead of the Standard Enumeration Areas (SEAs) that were used for Zambia, a vast country with a population density of about 19 inhabitants per square kilometre. This made it difficult for some potential participants who had to walk very long distances to get to the survey operations site. The inclusion of HIV testing in this survey may have had a negative impact on the participation rate as well; nonetheless, the HIV testing was administered at the end of the TB related procedures with a separate consent. Considering that the HIV testing was offered after the participants had already participated in the TB processes, we would therefore assume that this did not affect the participation rate in the TB survey significantly. Studies to explore the effect of inclusion of HIV testing on participation in TB prevalence surveys are therefore recommended for future surveys and in other settings.

Other countries which have conducted prevalence surveys before reported varying participation rates with some reporting higher [8,9,10] while others reported yet lower participation rates [11,12,13] than the Zambia survey.

Almost all of those who participated underwent the screening process through both the symptom screening questionnaire interviews and CXR, with only approximately 1% being screened by interview only. The sputum collection rate was 91%, that is, individuals who submitted at least one sputum sample out of those who were eligible for sputum examination. The proportion of survey participants eligible for sputum collection was higher than what was found in other African countries such as Ethiopia and Nigeria [8, 11].

The prevalence of bacteriologically confirmed MTB was 638 per 100,000 adult population in 2013–2014. This prevalence of tuberculosis is higher than currently reported by routine surveillance; for instance the notification rate for bacteriologically confirmed TB for the same period in the age group of 15 years and above was 173 per 100,000 population. Extrapolation of the burden of all forms of TB resulted in a national level prevalence of 455 per 100,000 population (range 386-578/100,000) for all age groups in Zambia. This is within the WHO prevalence estimates for all forms of TB for the same period, which was 338 (193–524) per 100,000 population, but with a narrower confidence interval.

The survey has also shown that there are wide variations in the burden of TB by location, sex, social economic status and age category. The prevalence of MTB was two to three times higher in the urban than in the rural areas; this was true for all the forms of TB, smear, culture and bacteriologically confirmed. The risk of TB was higher in males than females in the ratio of 2:1 for all forms; this is consistent with what is normally observed with notifications in data from the routine surveillance system in this group, and a previous review of routine data indicated consistently higher notifications of males than females over a period of more than ten years [4]. The survey has also demonstrated the importance of the TB/HIV co–morbidity, with the HIV positive having a 5 times higher risk of TB than their HIV negative counter parts. However, there were more participants with TB among the HIV negative individuals compared to the HIV negative, therefore underscoring the fact that high TB burden of HIV negative individuals in the communities is equally important.

In terms of socio-economic status using wealth tertiles; the risk of MTB was two times higher among the highest wealth tertiles than the lowest tertiles in the rural areas. However, the opposite was true for the urban areas, where the lowest income level reported two times more TB in the lowest income level than the highest income level.

This survey has also highlighted that MTB cases are likely being missed by routine passive case finding as most of the symptomatic cases not on treatment had actually even sought care for their symptoms. Active case finding strategies should be employed in order to improve case detection and more programmes to inform the general public and communities about the disease should be considered. More than 90% of TB patients identified during the survey had not been identified prior to the time of the survey; the reasons for this need to be further investigated, however, the fact that this was a population based survey, most of the patients identified could have been in the early stages of the disease and hence may not have felt the need to go to the health facilities to be investigated. On the other hand some of them had sought care at one point at the health facilities and had not been diagnosed with TB. This could be due to inadequate knowledge on the part of the health care providers, therefore, underscoring the urgent need to ensure that health care providers should be adequately trained in order for them to have a high index of suspicion in presumptive TB patients. There is a need to sensitize health workers on ensuring that they provide quality health care services so as to avoid missing cases which could have otherwise been picked. This presents a pool for perpetuation of the TB burden if it is left unchecked.

The cases of TB were largely occurring between the age group 25–44 years (51%) with the peak being around the 35–44 age bands; a group which is also economically viable and hence points to the TB burden having potential economic consequences on the micro and macroeconomic level [14]. This is an important area for future research to estimate the economic consequences of TB especially among the workforce in Zambia. The survey detected more cases in the age group similar to what is pertaining under routine surveillance [4].

Of the survey cases identified, 49 percent were smear negative, meaning that these cases could have been missed by routine case detection since it is normally done without culture identification. However, this finding may even be an underestimation because in some cases two culture results were not available in significant proportion for the individuals who were eligible for sputum submission. It is therefore important to scale up TB culture laboratory facilities and also investment in better point of care diagnostic approaches is required so that the chances of missing MTB cases are minimized.

Additionally, 62 percent of the smear positive cases were actually found not to be MTB as some of the cases were NTM. It is therefore recommended that smear positivity alone may not be adequate in the detection of TB patients especially when intensified case finding or active case detection strategies are employed. More specific point of care tests are required, so that only true MTB positive cases are put on anti-TB treatment. In fact, there are some studies that have shown that smear positivity is not a good indicator of MTB disease [8,15].

If only one of the symptoms (lasting two weeks or more) was used to screen potential cases; 84 percent, 57 percent and 53 percent of the survey cases would have missed by fever, cough and chest pain respectively. Fortunately, in this survey, all the three symptoms were used in combination or alone to come up with potential survey participants to submit sputum for laboratory investigation. Nonetheless; using symptoms alone; 39 percent of the survey cases could have gone undetected. The additional 104 out of the 265 cases were detected using chest x-ray screening. It is also likely that even the bacteriologically negative individuals with abnormal CXR could later develop TB as has been shown before [16]. Therefore, post-survey follow up of participants with abnormal CXR is highly recommended.

On the other hand, if only chest-x-ray screening alone was used to identify cases eligible for sputum submission; 17% of the survey cases would have been missed. The use of an elaborate symptom screening algorithm resulted in a more robust screening criteria hence minimising the possibility of missing cases in this survey. This is unlike other surveys, for example Pakistan [13] and Vietnam [12] used cough only]. It is therefore cardinal to use both symptom screening in combination with chest x-ray screening so as to optimize the case detection TB prevalence surveys. Missing of cases would lead to an underestimation of the prevalent cases in a given country.

Another important feature of the Zambian survey was the use of Xpert for all cases which were either smear positive or the culture results were inconclusive. This way; 17 additional cases were detected which could have otherwise gone undetected (Table 1). The Xpert was especially useful when the only available culture sample was contaminated and CXR was consistent with TB. The role of Xpert in national TB surveys needs to be further evaluated in other settings.

Through the use of digital systems, the usually anticipated data transcription errors were minimized through the use of pre-programmed electronic questionnaires, barcoding and scanning thereby leading to efficient and quality data collection. The digital data collection systems enabled daily monitoring of field operations and the survey teams to receive immediate feedback on data quality so that necessary improvements were made on a continuous basis with no or minimal disruption of field operations. This resulted in high quality data being collected and real time data checking. The application of technology and digital collection systems also reduced the time from data collection to reporting of results.

The TB prevalence in the adult population in Zambia in 2014 (638/100,000; range 502-774/100,000) is similar to what was found in Nigeria in 2014 (524/100,000; range 378-670/100,000), Myanmar in 2010 (613/100,000; range 502-748/100,000) and Cambodia in 2011 (831/100,000 range 707-977/100,000) but is higher than that of Ethiopia (277/100,000; range 208–347) and Pakistan (296.6/100,000 range 248.1–345.2/100,000) in 2011 [8, 9, 10, 11, 13]. This therefore shows that the burden of TB in Zambia, like in many low income countries, is very high requiring intensified and innovative TB control strategies [17] without which TB elimination may not be achieved [18].

The second TB prevalence survey after seven to ten years of intensive implementation of the National TB control program will be important to measure the difference in the disease burden and help assess whether or not the global and national target to reduce prevalence has been a success in Zambia.

### Limitations

It is acknowledged that national prevalence surveys have inherent limitations as information on paediatric TB (< 15 years old), extra pulmonary TB, smear and culture negative cases is not collected.

## Conclusion

The TB burden in Zambia is higher than previously estimated through routine surveillance. There is need to improve case detection rates especially among the identified hot spots in the sub-regions. This survey has also shown that digital systems are feasible. We have also demonstrated that TB surveys can integrate HIV testing so that the TB/HIV co-morbidity is better estimated. The application of digital data collection systems is recommended for future national TB prevalence surveys in order to make data collection more efficient and reduce time from data collection to results reporting.
